# Cultivable and metagenomic approach to study the combined impact of nanogypsum and *Pseudomonas taiwanensis* on maize plant health and its rhizospheric microbiome

**DOI:** 10.1371/journal.pone.0250574

**Published:** 2021-04-26

**Authors:** Parul Chaudhary, Priyanka Khati, Anuj Chaudhary, Damini Maithani, Govind Kumar, Anita Sharma

**Affiliations:** 1 Department of Microbiology, College of Basic Sciences and Humanities, G.B. Pant University of Agriculture and Technology, Pantnagar, Uttarakhand, India; 2 Crop Production Division, Vivekananda Parvatiya Krishi Anusandhan Sansthan, Almora, Uttarakhand, India; 3 School of Agriculture and Environmental Sciences, Shobhit University, Gangoh, Uttar Pradesh, India; 4 Crop Production Division, Central Institute for Subtropical Horticulture, Lucknow, Uttar Pradesh, India; South China Agricultural University, CHINA

## Abstract

In the present study we examined the effect of nanogypsum and *Pseudomonas taiwanensis* strain BCRC 17751on plant and soil health using conventional and metagenomics approaches. Soil physicochemical properties and agronomical parameters of maize plants were reported to be better when applied with nanogypsum and bacterial inoculum together. When compared to control a significant increase in total bacterial counts, nitrogen, phosphorus, potassium (NPK) solubilizing bacterial population and soil enzyme activities (fluorescein diacetate, alkaline phosphatase, dehydrogenase, β-glucosidase, arylesterase and amylase) was reported in treatments. The metagenomics studies revealed dominance of beneficial bacteria such as *Proteobacteria*, *Bacteriodetes*, *Planctomycetes*, *Acidobacteria* and *Nitrospirae* in treated soil. On the other hand some novel bacterial diversity was also reported in treated soil which was evident from presence of taxonomically unclassified sequences. Hence, it can be concluded that combined application of nanogypsum and *Pseudomonas taiwanensis* in maize help in improving the structure and function of soil which affects the plant health without causing any toxic effect. However, in situ validation of the prescribed treatment is required under field conditions on different crops in order to give maximum benefits to the farmers and the environment.

## Introduction

Agriculture system has observed dramatic changes since the time of its inception. It started with domestication of several wild plant species to small-scale traditional farming and then large scale intensive farming with the involvement of chemicals and hybrid seeds. Agriculture puts huge pressure on available resources like water, soil and biodiversity which is likely to increase in near future with an aim to feed ever growing human population globally. Thus, new technologies with positive impact on the environment are always in demand in agriculture sector to produce sufficient food. These technologies must be easily accessible and cost effective for the farmers. Application of bio-fertilizer has emerged out as an important holistic approach in agriculture to grow organic food and to maintain long term sustainability with no toxic effect on soil/ products **[1]**. Similarly, nanotechnology is also being tested in agricultural practices. To investigate the after effects of nanocompounds and/ or bioinoculants on soil/ environment, it is essential to study the soil quality, diversity, distribution and behaviour of microorganisms in soil habitats using a model cropping system. *Zea Mays* is generally main short term crops with good nutritive qualities. Moreover it is also used as a model crop for research purpose especially in the field of plant-microbe interactions **[2]**.

Plant growth promoting rhizobacteria (PGPR) belong to a group of useful bacteria that live in close association with plant rhizosphere and maintain plant growth either by defending them from environmental stress or diseases and/or by providing essential nutrients and hormones through various mechanisms **[3]**. PGPR are the best bioinoculants and may provide positive environment for the development of plants **[4]**. Some common PGPR viz. *Pseudomonas fluorescens*, *Bacillus* spp., *Pantoea agglomerans*, *Burkholderia* spp. etc. are reported to involve in solubilization of essential minerals, production of plant hormones and antimicrobials and developing resistance in plants **[5–7].**

In recent year, application of nanotechnology to boost agriculture production has received much attention **[8]**. Nanoparticles with less than 100 nm dimensions and due to some unique physical properties have found various applications in agriculture, food technology and environmental protection **[9]**. A significant amount of research based on NP concentration and their dose dependent relationships with plants has been carried out by number of researchers. Nanocompounds impose positive as well as negative impact on plant health **[10]**. Accessibility of low-cost nanomaterials is essential to enhance valuable use of nanotechnology in agriculture. Gypsum is an essential plant nutrient and found in soluble form of calcium and sulphur. It helps to improve physico-chemical properties of the soil and makes agriculture more sustainable and productive. Application of gypsum as a soil amendment recovers soil properties and improves water availability, quality of soil and enhances growth of alfalfa **[11]**. According to **Kumar and Thiyageshwari [12]** gypsum and nanogypsum help in reclamation of sodic soil by forming Ca^2+^ exchange complexes. **Parul [13]** observed that application of nanogypsum enhanced plant growth and microbial flora of the experimental soil. Practice of using nanocompounds in agriculture sector has not been yet made to a larger extent due to certain ecotoxicological effects and inconsistency in their performance. Therefore, application of mixture of nanocompounds and indigenous PGPR (as bioinoculant) in agricultural practices could be an excellent strategy to stimulate plant health, maintain soil health and to reduce the influence of toxic chemicals on agricultural produce **[14, 15]**.

Rhizospheric microbiome harbours higher microbial population than bulk soil and significantly affects quality of plant and soil. Microbial population of rhizospheric soil plays important ecological and physiological functions and maintains soil quality and habitability for plants. It enhances nutrient uptake, protects plants against pathogens, enhances abiotic stress tolerance and produces number of secondary metabolites and leads to enhanced plant growth **[16]**. Several rhizospheric microbes are known to secrete extracellular enzymes like phosphatases, dehydrogenases, proteases and lipases to obtain carbon, nitrogen, energy and other essential nutrients from the mineralization of complex polymers. Soil enzymes act as specific indicator of soil health/ fertility and represent total microbial activities of the soil **[17]**. They play vital role in recycling of nutrients and organic matter degradation in soil and respond quickly towards any change in the soil management and environmental conditions. Microbes constitute a biological entity of the soil and their diversity could be studied using culturable and unculturable methods. Only a small proportion of the microorganisms can be studied using culture-based methods **[18]**. Molecular techniques targeting 16S ribosomal DNA genes have provided better opportunity to evaluate microbial population in a system against culture-based methods. Illumina based approaches provide details of a microbial community more precisely **[19]**. It provides significant information in the changes of microbial composition and structure. The plan of this study was to analyze the growth of maize plant and microbial diversity of rhizospheric soil after the treatment of nanogypsum and *Pseudomonas taiwanensis* using culturable and metagenomic approaches.

## Materials and methods

### Bio-inoculant and nanocompound used

Bacterial culture (PC1-*Pseudomonas taiwanensis* strain BCRC 17751, accession number MK106029) used in the present study was recovered from an agriculture field of GBPUA&T, Pantnagar. Used bioinoculant in this study were found positive for a variety of plant growth promoting activities like siderophore and IAA production and solubilization of phosphate, potassium and zinc **[6]**. Nanogypsum (13 nm size, polydispersion index 0.204) with 98% purity was provided by Department of Agronomy, GBPUA&T, Pantnagar. All the other chemicals were purchased from SRL Laboratories Pvt. Ltd. India and Hi media.

### Experimental design

The soil for pot experiment was collected from Crop Research Centre of GBPUA&T, Pantnagar, where agricultural plots of different crops were treated with nanocompound for six years. The altitude of the region is about 243.84 meters above sea level which comes under subtropical climatic zone. Pot experiment with maize was conducted in June, 2018 at the Departmental net house. Twelve pots were filled with 2 kg fine sieved soil. Completely Randomize Design (CRD) was used for experimental design with three replicates per treatment. Eight seeds were sown in each pot at the depth of 5 cm. Different treatments used are follows: control (without bacteria and nanogypsum), PC1 (*Pseudomonas taiwanensis*), NG (nanogypsum), PC1+NG (*Pseudomonas taiwanensis* along with nanogypsum) **(Table 1)**.

**Table 1:** Detail of the treatments.

Treatments	Description
**AC**	Absolute control
**PC1**	*Pseudomonas taiwanensis*
**NG**	Nanogypsum
**PC1+NG**	*Pseudomonas taiwanensis*+Nanogypsum

### Seed bacterization and preparation of bacterial inoculum

Seeds of maize (variety ‘DH296’) were washed thoroughly with tap water. Surface sterilization of healthy seeds was done for 2 minutes in 0.1% mercuric chloride solution which was further rinsed three times with sterilized distilled water to remove the residual traces of HgCl_2_. Seeds were soaked in overnight grown bacterial culture. Single seed received a population of 3×10^7^ CFU along with nanogypsum (50 mg L^-1^). Control seeds did not have either bacterial culture or nanocompound. Seeds were further incubated at 70 rpm at 25°C for 15 minutes on a rotary shaker in the flasks. Coated seeds were dried in laminar bench for 2h and eight seeds per pot were sown. Pots were watered daily as per the moisture requirement.

### Soil and plant sample collection

Rhizospheric soil from the roots of plants was collected by gentle shaking and mixed to generate a representative composite sample after 30 days of the pot experiment. Soil samples were kept at -20^0^ C to analyze soil physicochemical analysis, indicator enzymes, CFU counts and microbial community analysis. Physico-chemical parameters like soil pH, available nitrogen, phosphorus, potassium, soil organic carbon and nitrate nitrogen were analysed using HiMedia kit.

### Measurement of plant health parameters

Germination of seeds, plant length, number of leaves, total chlorophyll, carotenoid, total sugar, phenol, protein and antioxidant enzymes (catalase, peroxidase and superoxide dismutase) were calculated after 30 days of the experiment.

### Chlorophyll estimation

Maize leaves were thoroughly cleaned with distilled water to avoid any contamination on surface. Fifty mg of leaves in test tube were mixed with 10ml DMSO (dimethyl sulfoxide). Tubes were incubated in water bath at 60°C for 3h or till the leaves become colourless. The extract was filtered and maintained at room temperature. Absorbance of the leaf extract was taken at 645 and 663 nm with DMSO as control **[20]**. The absorbance of same leaf extract was measured at 470nm for calculation of carotenoid content **[21]**.

### Total sugar estimation

Fresh leaves of maize were dried at 80°C for 48h in hot air oven. Dried leaves (0.1g) were crushed with 3ml of 80% ethyl alcohol, then boiled and centrifuged at 1000rpm for 15 min. Four ml of Anthrone reagent was added to 1ml leaf extract and boiled for 10 min. Reading was taken at 620nm and sugar content was calculated using standard curve of glucose **[22]**.

### Protein estimation

Fresh leaves were collected, washed and crushed gently in 5ml of Tris-cl (0.2M, pH-8) to form fine slurry and then centrifuged at 10,000 rpm (4°C, 20 min). Supernatant was transferred to fresh tube and stored for further use at 4°C. Twenty microlitres of supernatant were added to 280μl of extraction buffer and 3ml Coomassie Brilliant Blue (CBB) G-250. Obtained mixture was kept at 37°C for 5 min and absorbance was taken at 595nm against a blank **[23]**.

### Estimation of total phenolic content

Fresh maize leaves (200mg) were mixed in ice cold methanol (800μl of 95%) in a pestle and motor and store in for 48h at room temperature. Mixture was centrifuged at 10,000rpm for 5 min. The supernatant was used to estimate the phenolic content by Folin-Ciocalteu method and gallic acid was used as standard **[24]**.

### Analysis of antioxidant enzymes

#### Catalase activity (CAT)

Catalase activity was determined according to **Chandlee and Scandalios [25]**. Reaction was started by adding enzyme extract (100μl) to the reaction mixture (3ml), with 100mM sodium phosphate buffer (pH-7) and 10mM H_2_O_2_ (0.1ml). Decline in reading was checked at 230nm for 3min in a visible spectrophotometer. Assay mixture devoid of enzyme extract used as control. Enzyme activity was estimated by using extinction coefficient (39.4mM ^-1^ cm^-1^) and expressed as disintegration of 1mM H_2_O_2_ min^-1^.

#### Peroxidase activity (POD)

For this 3ml reaction mixture containing 0.1ml of the enzyme extract, 0.4ml of pyragallol in phosphate buffer and 0.5ml of H_2_O_2_ were added in cuvette and change in absorbance was recorded at 420nm for a period of 3min. Control does not contain any enzyme extract. Enzyme activity was calculated by using 26.6mM^-1^ cm^-1^extinction coefficient **[26]**.

#### Superoxide dismutase activity (SOD)

SOD activity was estimated on the basis of Nitroblue tetrazolium chloride (NBT) inhibition, which was measured spectrophotometrically at 560nm **[27]**. The reaction mixture for SOD activity was prepared using riboflavin (75mM), 100mM phosphate buffer (pH-7.5), methionine (200mM), 3 mM EDTA and 100μl of enzyme extract. SOD enzyme activity was expressed as units of enzyme g^-1^ FW.

### Enumeration of total bacterial and NPK dissolving CFU count

Enumeration of total bacterial population and NPK dissolving bacteria from the experimental soil was performed after 30 days of the pot trial. Ashby, Pikovskaya and Aleksandrow media were used to count nitrogen fixing (*Azotobacter*), phosphate and potassium solubilising bacteria respectively. Plates were further incubated at 28^0^ C for 3–4 days.

### Enzyme activities of soil

#### FDA hydrolysis

1g of soil was mixed with sodium phosphate buffer (50ml, pH-7.6) and FDA solution (0.5ml) was added to the mixture and incubated in shaker (1h at 24°C). The reaction was terminated with 2ml acetone. Centrifugation of suspension was done at 8000rpm for 5min and supernatant was filtered through filter paper No.2 (Whatman). Reading was taken at 490nm and enzyme activity was expressed as μg fluorescein released g^-1^ dry soil h^-1^ **[28]**.

#### Dehydrogense activity

Dehydrogenase activity was estimated according to **Casida et al. [29]** using TTC (Triphenyl tetrazolium chloride) solution. To 5 g soil 5mL of TTC (2g in 100mL, 0.1 M Tris buffer, pH 7.4) was added. Twenty-five ml of acetone was used to extract triphenyl formazan (TPF) and centrifuged at 4500rpm for 10min at 4°C. Supernatant was filtered through Whatman filter paper No. 1 and absorbance was recorded at 485nm. Dehydrogenase activity was expressed as μg TPF 5g^-1^ dry soil 8h^-1^.

#### Alkaline phosphatase activity

To 1 gram soil, 250μl of toluene, 4ml MUB buffer and 1ml p-nitrophenyl phosphate (pNpp) were added in test tubes. Tubes were incubated at 37^0^ C for 2h. After incubation, Tris buffer (4ml, 0.1M, pH -12) and CaCl_2_ (1ml) were added to the mixture. The suspension was left to develop colour and filtered before recording the absorbance at 400nm. Enzyme activity in soil sample was expressed as μg pNP released g^-1^ dry soil h^-1^ **[30]**.

#### β-glucosidase activity

To 1 g dry soil, 0.25 ml of toluene, 1ml of p- nitrophenyl-β-D-glucoside (pNPBG) and modified universal buffer (4ml, pH 6.0) were added in test tube. Tubes were incubated at for 1h at 37°C. CaCl_2_ (1ml, 0.5 M) and Tris buffer (4ml with pH-12) were added in test tube. The suspension was left to develop colour and filtered before recording the absorbance at 410nm. Enzyme activity was expressed as μg pNP released g^-1^ dry soil h^-1^ **[31]**.

#### Amylase activity

1gram of soil sample mixed with 2.5 ml of phosphate buffer (pH 6) and 1% starch (1ml) was added. Test tubes were placed in shaker for 6h at 30^0^ C, centrifuged for 10 min at 12000 rpm. After adding 1ml of DNS reagent to 1ml of supernatant, tubes were kept for 5 min in boiling water bath. Absorbance was measured at 540 nm after adding 3 ml distilled water **[32]**.

### Arylesterase

After adding 2 ml MUB and 0.5ml pNPA (200 mM) to 1g soil, tubes were vortexes and placed in water bath at shaking condition for 1h. Mixture was centrifuged at 4°C for 5 min at 6500rpm. 1ml of supernatant was transferred to a new test tube and 2ml n-hexane was added. Aqueous layer (0.5ml) was taken to which 0.5ml (1M) sodium hydroxide and 4 ml double distilled water were added. Reading was taken at 400nm and enzyme activity was expressed as μg pNP released g^-1^ dry soil h^-1^ **[33]**.

### Metagenomic sequencing through NGS

Two soil samples were processed for metagenomic analysis. Two hypervariable region (V3-V4) regions of 16S rRNA gene were amplified using primers (341F-5’CCTACGGRRBGCASCAGGKVRVGAAT; 785R-5’GGACTACNVGGTWTCTAATCC). After the purification of amplicons, paired-end sequencing was done on an Illumina Mi-Seq platform. OTUs were identified from all the reads using QIIME software package and a representative sequence for each OTU was also constructed.

### Statistical analysis

The values of above parameters were expressed as mean ± SD (standard deviation). One way ANOVA was carried out using SPSS software to assess significant variation between the means of different treatments.

## Results

### Agronomical parameters of maize crop

Seed germination was invariably high in treated soil and highest percent (97%) seed germination was recorded in combined treatment of nanogypsum and *Pseudomonas taiwanensis* (PC1+NG). Seed germination in other treatments like PC1, NG and control was 90.29%, 87.49% and 69% respectively. Average plant height for treated samples was 58.26 cm in PC1+NG treatment followed by 46.23 cm, 42.83 cm and 30.66 cm in NG, PC1 and control respectively (**Table 2**). Higher root length in all the treated plants was recorded over control. Root length in PC1+NG treatment was 32.40 cm followed by NG (25.06 cm), PC1 (24.43 cm) and control (15.13 cm) treatments. Leave area number of leaves were also high in treated plants as compared to control **(Table 2)**.

**Table 2:** Effect of nanogypsum and *Pseudomonas taiwanensis* on agronomical parameters of maize treated plants.

Treatments	Germination (%)	Plant height (cm)	Root length (cm)	Number of leaves	Leaf area (cm^2^)	Total Chlorophyll (mg g^-1^)	Carotenoid (mg g^-1^)
**AC**	69.00 ± 3.60^a^	30.66 ± 1.52^a^	15.13 ± 0.23^a^	5.44 ± 0.50^a^	12.77 ± 1.92^a^	2.16 ± 0.12^a^	0.099±0.008^a^
**PC1**	90.29 ± 2.37^b^	42.83 ± 2.25^b^	24.43 ± 0.51^b^	6.11 ± 0.19^ab^	27.57 ± 1.28^c^	3.49 ± 0.19^b^	0.148±0.007^c^
**NG**	87.49 ± 4.16^b^	46.23 ± 1.07^c^	25.06 ± 1.05^b^	6.10 ± 0.17^ab^	22.20 ± 0.46^b^	3.52 ± 0.22^b^	0.125±0.004^b^
**PC1+NG**	97.22 ± 2.40^c^	58.26 ± 0.64^d^	32.40 ± 2.19^c^	6.65 ± 0.65^b^	35.16 ± 1.25^d^	4.30 ± 0.11^c^	0.177±0.011^d^

Means in each column followed by the same letter were not significantly different (P ≤ 0.05) as determined by one-way ANOVA and Duncan’s Multiple Range Test (DMRT). Values were the means of three replications ± SD.

Chlorophyll content was reportedly high in treated plants than the control and the pattern observed was: PC1+NG >NG > PC1>control which was in the range of 4.30, 3.52, 3.49 and 2.16 mg chlorophyll g^-1^respectively. Similarly, carotenoid content was also high in all the treatments in comparison to control. An increase of 1.77, 1.49 and 1.26 fold in carotenoid content was observed in PC1+NG, NG, PC1 treatments respectively over control **(Table 2)**. Highest sugar content (43.92 mg g^-1^) was observed in combined treatment of PC1+NG. Sugar content in other treatments like PC1, NG and control was 36.89, 37.83 and 23.16 mg g^-1^ respectively. For total protein PC1, NG and PC1+NG treatments showed 1.24, 1.21 and 1.38 fold increase respectively over control. Content of phenolics was better in combined treatment of bioinoculant and nanogypsum in comparison to other treatments and was 2.61, 2.33, 2.30 and 1.42 mg g^-1^ in PC1+NG, PC1, NG and control respectively (**Table 3)**. Application of nanogypsum along with *Pseudomonas taiwanensis* also improved innate response of the plants by enhancing the activities of CAT, POD and SOD enzymes. PC1, NG, PC1+NG treatments showed 1.11, 1.08 and 1.52 fold increase respectively in CAT activity as compared to control. Similarly, the level of POD was 1.47, 1.29 and 1.71 fold higher in PC1, NG, PC1+NG treatments respectively than control. SOD activity was high in all the treated samples as compared to control. Pattern of SOD activity was PC1+NG > PC1 >NG with (1.56) > (1.26) > (1.23) fold increase respectively over control **(Table 3)**.

**Table 3:** Effect of nanogypsum and *Pseudomonas taiwanensis* on biochemical parameters and antioxidant enzymes of maize treated plants.

Treatments	Total sugar (mg g^-1^)	Protein (mg g^-1^)	Phenol (mg g^-1^)	Catalase (μmol min^-1^ mg^-1^ protein)	Peroxidase (μmol min^-1^mg^-1^ protein)	SOD (μmol min^-1^mg^-1^ protein)
**AC**	23.16 ± 1.11^a^	12.37 ± 0.37^a^	1.42 ± 0.02^a^	10.48 ± 0.36^a^	42.42 ± 0.39^a^	10.85 ± 0.12^a^
**PC1**	36.89 ± 1.50^b^	15.35 ± 0.33^b^	2.30 ± 0.14^b^	11.66 ± 0.28^b^	62.45 ± 0.51^c^	13.70 ± 0.34^b^
**NG**	37.83 ± 1.58^c^	15.07 ± 0.19^b^	2.33 ± 0.04^b^	11.35 ± 0.13^b^	54.78 ± 0.38^b^	13.40 ± 0.23^b^
**PC1+NG**	43.92 ± 1.00^d^	17.08 ± 0.26^d^	2.61 ± 0.01^c^	15.96 ± 0.29^c^	72.68 ± 0.72^d^	17.00 ± 0.12^c^

Means in each column followed by the same letter were not significantly different (P ≤ 0.05) as determined by one-way ANOVA and Duncan’s Multiple Range Test (DMRT). Values were the means of three replications ± SD.

### Soil physicochemical analysis

Physicochemical analysis of the soil samples was performed qualitatively to assess the nutrient status and soil health under various treatments. The pH values of different treatments were quite variable from control. Highest pH (7.8) was observed in PC1+NG treated soil. Maximum level of soil organic carbon (0.750–1.00 Kg ha^-1^) was observed in PC1+NG treatment. Level of available phosphate was higher in PC1+NGtreatment (56–73 Kg ha^-1^), whereas in control and nanogypsum treatment medium level was reported. Available potassium was medium in all the treated soils (112–280 Kg h^-1^) and control had lowest level (>112) of available potassium. Level of ammonical nitrogen and nitrate nitrogen was also high in all treated soil samples than control **(S1 Table)**.

### Total bacterial and NPK count of the treated soil

Total bacterial counts were observed to be improved in all treatments in comparision to control. Order of bacterial counts for different treatments was 2.34×10^6^, 2.18×10^6^ and 2.14×10^6^ for PC1+NG, PC1 and NG respectively which was significantlly higher than control (2.10×10^6^). Nitrogen fixing bacterial counts were high in combined treatment of nanogypsum and bioinoculant. Control had lowest population of N_2_ fixers where as PC1+NG, PC1 and NG treatments had 8.36×10^5^, 7.10×10^5^ and 7.06×10^5^ population respectively and differed significantlly from control (5.73×10^5^). Number of phosphate and potassium solubilizing bacteria in PC1+NG, PC1 and NG treated soil was in the range of 8.86×10^5^, 7.76×10^5^ and 7.63×10^5^ cfu g^-1^and 7.60×10^5^, 6.03×10^5^ and 6.00×10^5^ cfu g^-1^ respectively **(Table 4)**.

**Table 4:** Effect of nanogypsum and *Pseudomonas taiwanensis* on bacterial count (CFU) of soil under maize cultivation.

Treatments	Total bacteria	N fixers	P solubilizers	K solublizers
**AC**	2.01×10^6^ ± 6.02^a^	5.73×10^5^ ± 6.42^a^	6.10×10^5^ ± 2.64^a^	4.63×10^5^ ± 4.04^a^
**PC1**	2.18×10^6^ ± 3.00^b^	7.10×10^5^ ± 6.00^b^	7.76×10^5^ ± 3.51^b^	6.03×10^5^ ± 4.04^b^
**NG**	2.14×10^6^ ± 7.02^b^	7.06×10^5^ ± 5.50^b^	7.63×10^5^ ± 5.13^b^	6.00×10^5^ ± 5.00^b^
**PC1+NG**	2.34×10^6^ ± 4.04^c^	8.36×10^5^ ± 4.04^c^	8.86×10^5^ ± 8.50^c^	7.60×10^5^ ± 3.60^c^

Means in each column followed by the same letter were not significantly different (P ≤ 0.05) as determined by one-way ANOVA and Duncan’s Multiple Range Test (DMRT). Values were the means of three replications ± SD.

### Soil enzyme activities

FDA can be used as an indicator to check soil health as it represents overall microbial activities of the soil and acts as the substrate of lipase, protease and esterase. PC1+NG treatment had highest activity (34.54 μg fluorescein g^-1^ h^-1^) followed by NG (27.29 μg fluorescein g^-1^ h^-1^) and PC1 (26.29 μg fluorescein g^-1^ h^-1^) treatments which was significantly better than control (15.00 μg fluorescein g^-1^ h^-1^). Maximum dehydrogenase activity (4.73μg TPFg^-1^ h^-1^) was observed in PC1+NG treatment which was also significantly better than the control (2.84μg TPFg^-1^ h^-1^). Dehydrogenase activity of PC1 and NG treated soil was 3.84 and 3.74 μg TPFg^-1^ h^-1^ respectively. Alkaline phosphatase activity was highest in combined treatment of nanogypsum along with bioinoculant. Level of phosphatase activity in different treatments like PC1+NG, PC1 and NG was 156.83, 133.33 and 117.67 μg PNP g^-1^ h^-1^ respectively. β-glucosidase activity was maximum (187.17 μg h^-1^) in combined treatment of PC1+NG where the activity in PC1 and NG treated soil was 160.17 and 164.50 μg h^-1^ respectively. Higher glucosidase activity could be related to higher population of cellulose degraders in the treated soil. Similarly, amylase activity was maximum (108.67 μg h^-1^) in PC1+NG treated soil followed by NG (91.66 μg h^-1^) and PC1 (64.66 μg h^-1^). Enzyme activity was significantly high in treated soil than control (50.50 μg h^-1^). Order of arylesterase activity was 43.66, 37.33, 31.12 and 17.11 μg h^-1^ in PC1+NG, NG, PC1 and control soil respectively. Enhanced level of enzyme activity is also an indicator of improved microbial activity of the soil which is due to recycling of nutrients by enzymes **(Table 5)**.

**Table 5:** Effect of nanogypsum and *Pseudomonas taiwanensis* on enzyme activities of the rhizospheric soil of maize.

Treatments	Fluorescein diacetate (μg g^-1^ dry soil h^-1^)	Dehydrogenase (μg 5g^-1^ soil 8h^-1^)	Alkaline Phosphatase (μg g^-1^ soil h^-1^)	β-Glucosidase (μg g^-1^soil h^-1^)	Amylase (μg 2g^-1^soil h^-1^)	Arylesterase (μg g^-1^soil h^-1^)
**AC**	15.00 ± 1.25^a^	2.84 ± 0.18^a^	56.33 ± 3.21^a^	85.66 ± 2.46^a^	50.50 ± 2.78^a^	17.11 ± 1.17^a^
**PC1**	26.29 ± 1.20^b^	3.84 ± 0.16^b^	133.33 ± 3.63^c^	160.17 ± 3.61^b^	64.66 ± 2.08^b^	31.12 ± 1.70^b^
**NG**	27.29 ± 0.64^b^	3.74 ± 0.07^b^	117.67 ± 3.51^b^	164.50 ± 4.67^b^	91.66 ± 4.01^c^	37.33 ± 2.02^c^
**PC1+NG**	34.54 ± 1.25^c^	4.73 ± 0.16^c^	156.83 ± 4.42^d^	187.17 ± 3.17^c^	108.67 ± 1.52^d^	43.66 ± 0.88^d^

Means in each column followed by the same letter were not significantly different (P ≤ 0.05) as determined by one-way ANOVA and Duncan’s Multiple Range Test (DMRT). Values were the means of three replications ± SD.

**Fig 1:**
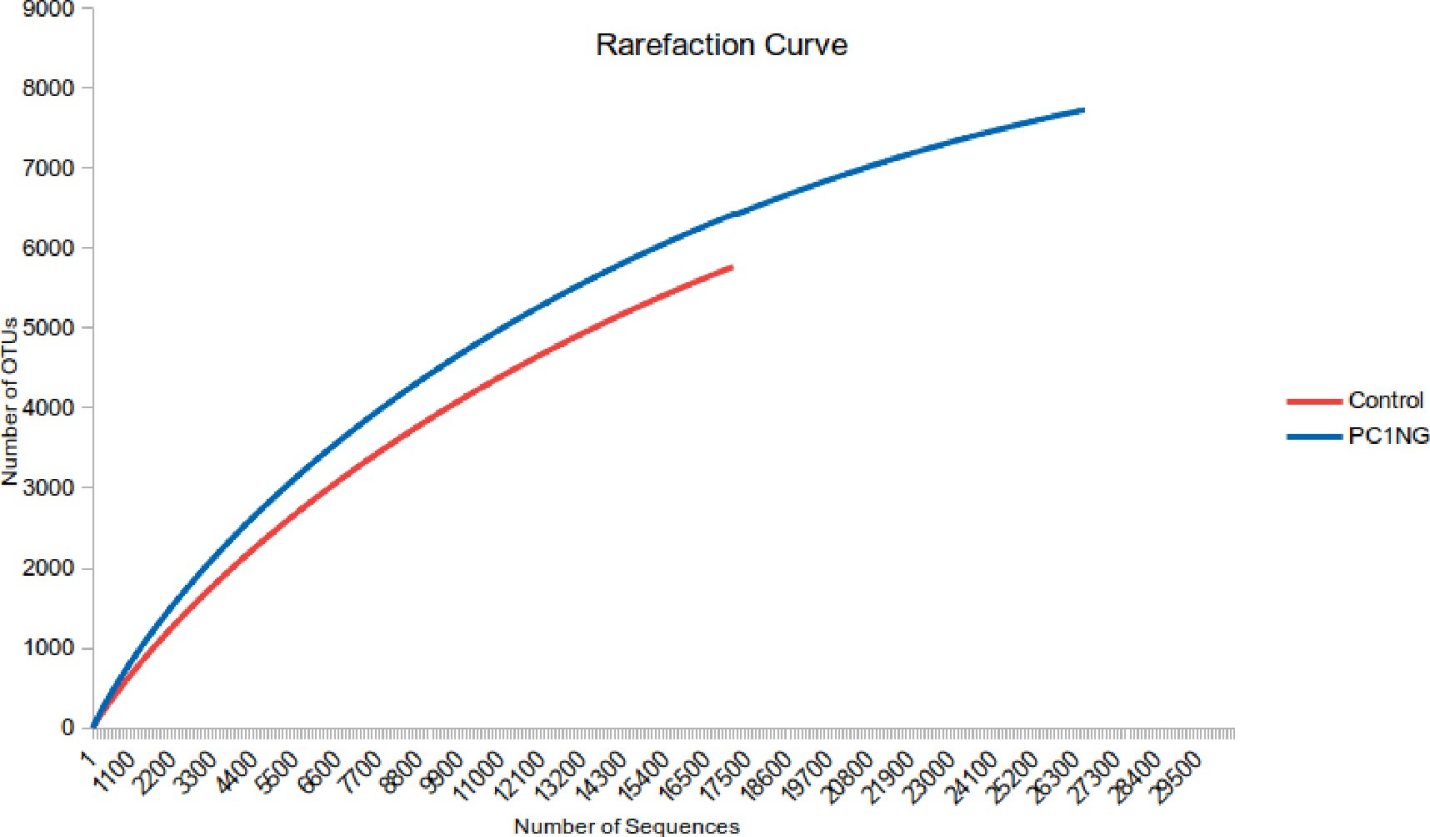
Rarefraction curve showing OTUs of control and treated soil sample under pot condition.

Soil under combined treatment (PC1+NG) showed highest bacterial counts and enzyme activities hence the same soil sample and control was used for metagenome sequencing to study the microbial diversity and functionality into deeper level. Diversity of bacterial species in the soil samples was analyzed using a series of statistical indices like ACE, Chao1, Shannon and Simpson. Shannon index represents evenness and richness of the species. Shannon diversity index for treated soil was 8.18 which were higher than the control (6.89). Rarefraction curve was used to determine species composition in both the samples. Steeper slope was observed for treated sample (PC1+NG) as compared to control which indicated species richness in treated sample over control **(Fig 1)**.

**Fig 2:**
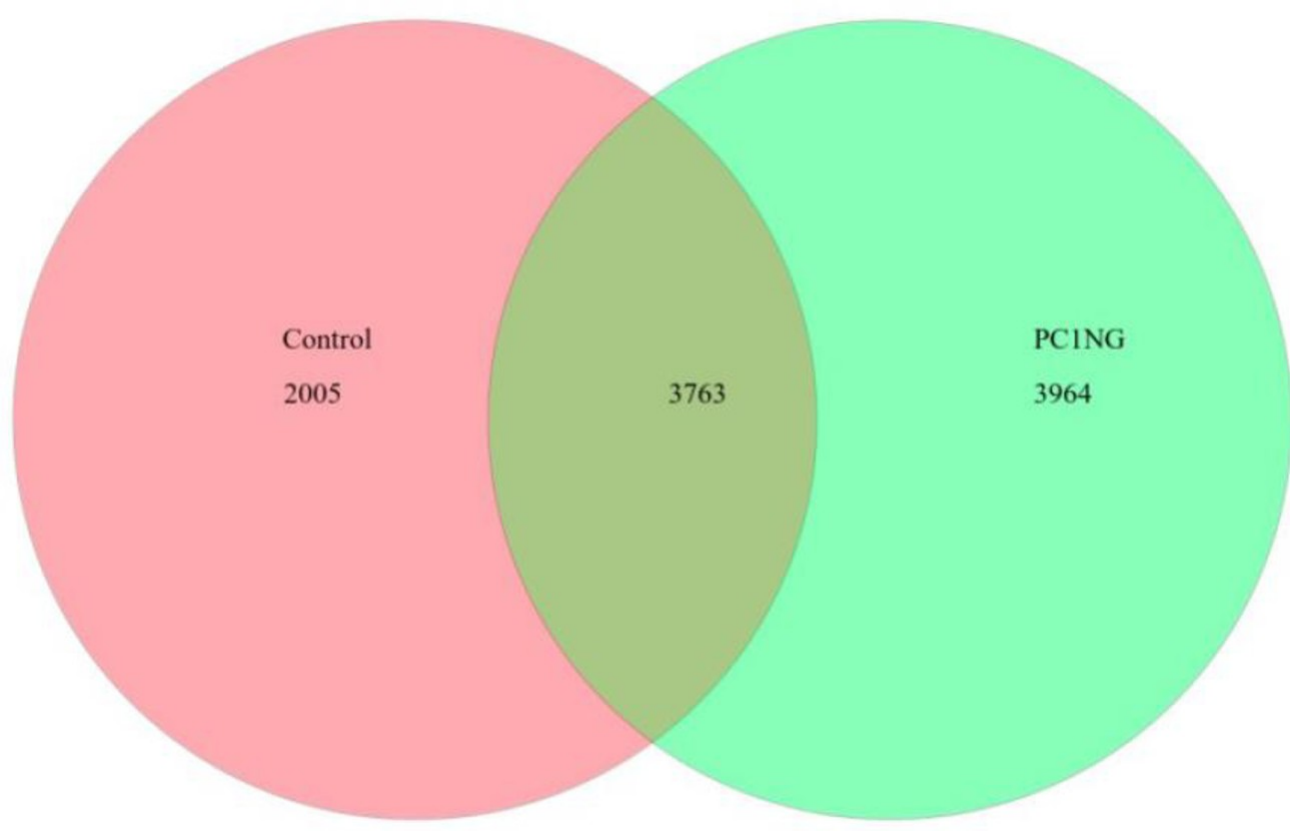
OTU Venn diagram of control and treated soil samples.

Total number of bacterial OTUs in both the soil samples was 9732. Out of which, 3763 OTUs were common in control and treated soil. A sum of 2005 and 3964 bacterial taxa were observed in control and treated sample respectively **(Fig 2)**. The sequencing data was submitted to SRA, NCBI with accession number PRJNA635642 and PRJNA635760 **(S2 Table)**.

### Predominance of taxonomic composition of bacteria at different level

**Fig 3:**
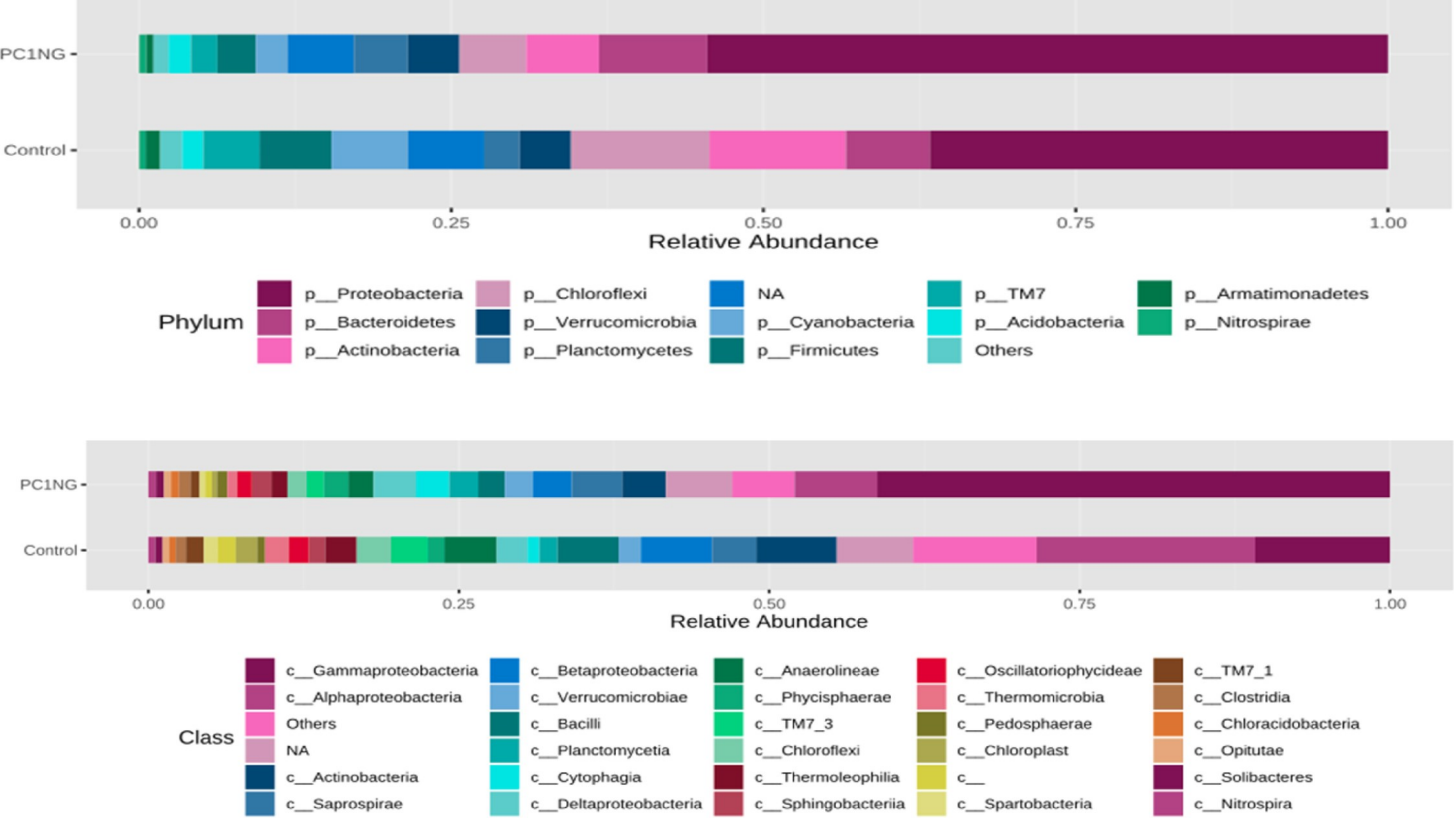
Stacked bar plots representing phylum and class distribution in control and treated soil sample.

**Fig 4:**
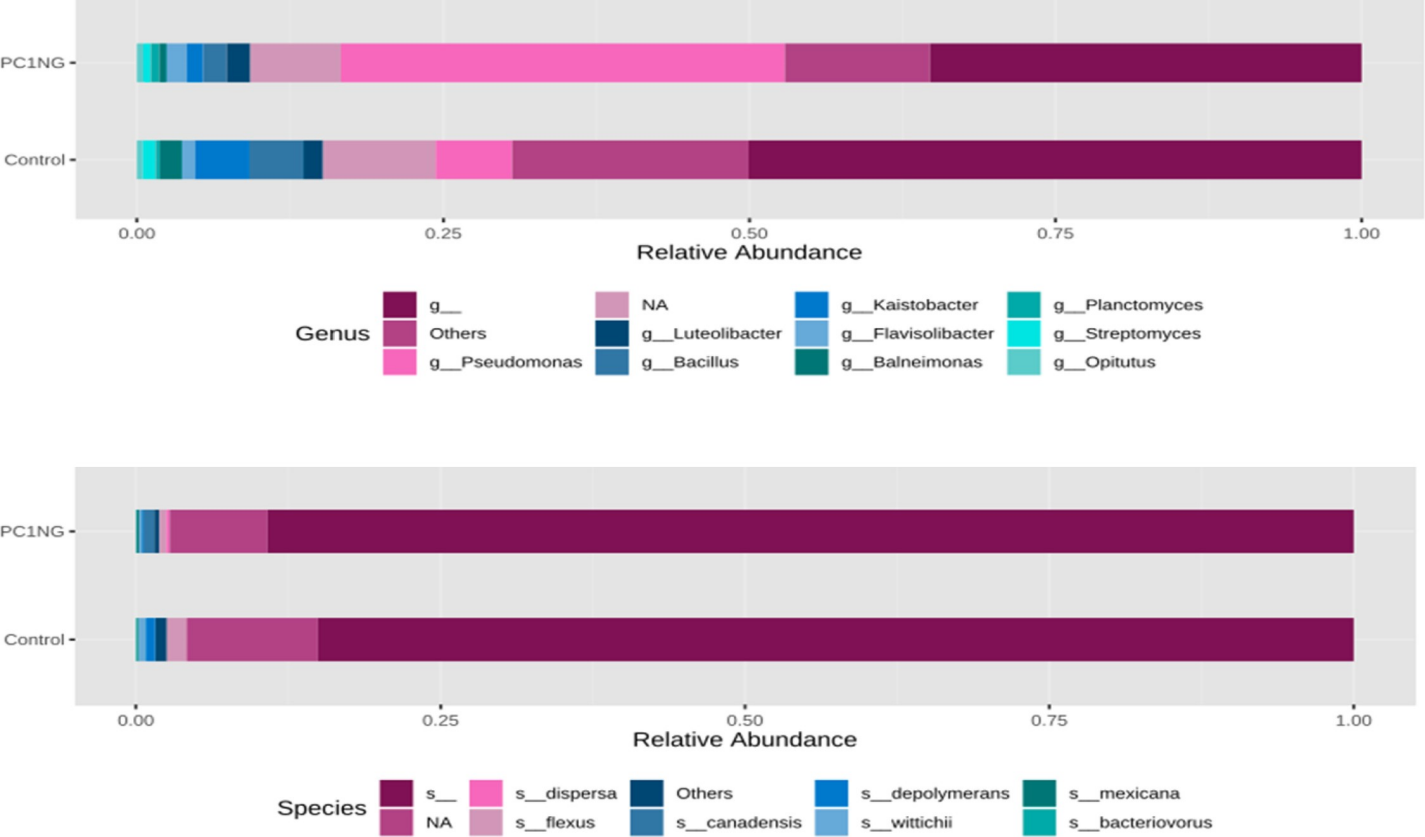
Stacked bar plots representing genus and species distribution in control and treated soil sample.

Soil sample treated with PC1+NG showed abundance of *Proteobacteria* (54%), *Bacteriodetes* (8.65%), *Planctomycetes* (4.34%), *Acidobacteria* (1.81%) and *Nitrospirae* (0.66%) as compared to control which showed 36%, 6.72%, 2.85%, 1.66% and 0.55% of the same bacterial genera respectively **(Fig 3).** Most abundant classes in treated and control soil samples were *α-proteobacteria* (control-17.62%, PC1NG-6.63%), *Gamma proteobacteria* (control-10.84%, PC1NG 41.30%), *Actinobacteria* (control-6.46%, PC1NG-3.55%), *Beta proteobacteria* (control-5.71%, PC1NG-3.14%), *Bacilli* (control-4.19, PC1NG-2.03%), *Saprospirae* (control-3.55%, PC1NG-4.03%) and *Delta proteobacteria* (control-2.48, PC1NG-3.41%) **(Fig 3)**. Relative abundance of genera like *Pseudomonas*, *Luteolibacter*, *Flavisolibacter*, *Opitutus* and *Planctomyces* was more in treated soil (36.27%, 1.93%, 1.64%, 0.55% and 0.70% respectively) than control. Based on the relative abundance, most of the bacterial species remained unidentified and could not be assigned to any group/ class in both the samples. Few species were identified as *flexus*, *depolymerans*, *wittichii*, *bacteriovorus*, *maxicana*, *dispersa* and *candensis* **(Fig 4)**.

## Discussion

Present study showed improvement in maize plant health and rhizospheric microbiome under the combined treatment of nanogypsum and *Pseudomonas taiwanensis*. The combined application also triggered faster seed germination in comparison to control. Improved seed germination in the presence of nanogypsum could be related to water retention property of nanogypsum which helps in regulating water channels and permeability of water in the seeds **[34]**. **Shinde et al. [35]** have reported enhanced seed germination in maize under the treatment of magnesium hydroxide (500ppm). Increased plant and root length, leaf number and area were also observed in treated plants over control. Similarly, **Khati et al. [36, 37]** reported improved seed germination, plant and root length, leaf area, chlorophyll and carotenoid content in maize plant under the treatment of nanozeolite and nanochitosan. Application of nanochitosan enhanced the growth of *Pantoea agglomerans* and *Pseudomonas taiwanensis* **[38]**. In this study, we observed that nanogypsum supported the growth of beneficial rhizospheric microbes through enhanced uptake of different nutrients required for plant growth and development **[39]**. Nanoparticles invariably chelate essential nutrients from the soil and release them slowly to support plant/ root growth for longer period. Improved plant length can be related to better availability of gibberrellic acid, indole acetic acid and phosphate by bioinoculants **[40, 41]**. Metagenomic study of maize rhizosphere under the treatment of nanocompounds showed improved microbial community and soil health **[42]**. Application of silver nanoparticles is reported to enhance agronomical and biochemical content in wheat plants **[43]**. Application of *Bacillus subtilis* and *Pseudomonas sp*. was also reported to improved plant/root length and yield of maize crop **[44]**.

Photosynthesis is the necessary physiological function of the plants to establish their efficiency. Our results revealed the significant increase in photosynthetic pigments in treated maize plants over control due to increase in chlorophyllase enzyme, stomatal conductance and chlorophyll fluorescence **[45]**. **Venkatachalam [46]** reported that zinc oxide nanoparticles improved the growth of cotton plants, chlorophyll, carotenoid content and total biomass over control.

In the present study total sugar, protein and phenolic content were also high in treated maize plants which may be related to positive and protected mechanisms shown by nanogypsum and *Pseudomonas taiwanensis*. **Mahakham [34]** also reported that nanoprimed seeds show enhanced sugar content because of improved activity of α amylase. Application of nanochitosan and chitosan also enhanced the level of phenolic compounds by 24% and 20% respectively in tea leaves **[47]**.

We report higher activity of antioxidant enzymes in combined treatment of nanogypsum and *Pseudomonas taiwanensis* in maize leaves. The antioxidant enzymes are well known shields for plants under stress condition. **Siddaiah et al. [48]** also observed increases in SOD activity when pearl millet seeds treated with CNP over control. **Sandhya et al. [49]** observed that application of *Pseudomonas* spp. helped in alleviating drought stress in maize.

Functional and taxonomical structures of microbial communities of the soil are influenced by type of flora and physicochemical characteristics of the soil **[50]**. The type of microflora also modulates the availability of nutrients in soil, which further affects the plants growth. Increase in pH, available phosphorus, nitrogen and potassium in treated soil is good for bacterial population directly and indirectly which can further support plant growth. It could be related to phosphate and potassium solubilizing efficiency of the bioinoculant used in the experiment. Micronutrients play a crucial role in the functioning of plants. They provide healthy environment for the growth of beneficial microbial population in the rhizosphere **[51]**. Nanoparticles also enhance nutrient utilizing efficiency of the plants by releasing nutrients according to their requirements which prevent conversion of nutrients to unavailable forms. Increase in NPK level of the soil is positively correlated to the presence of NPK dissolving bacterial population. Application of nanogypsum along with bioinoculant enhanced bacterial count in the soil. **Chai et al. [52]** reported that application of nano SiO_2_ also improved the population of NPK solubilizing bacteria, but zinc oxide nanoparticles decreased bacterial population due to the uptake of free ions. Similarly, application of PGPR and nanosilicon dioxide not only improved the plant health of maize but also supported the microbial dynamics in rhizospheric soil **[53]**.

Soil nutrients and enzyme activities are closely related. Major micro and micronutrients in soil are regulated by the soil enzymes, which are produced by native microbial population. Soil enzymes are known as early indicator of soil health are very sensitive to any change in the rhizospheric zone. Different soil enzymes are used as indicator to detect any change in the soil quality, microbial community and diversity **[54]**. Enzymes represent the functioning of the entire microbial community in an ecosystem. Significant increase in FDA, dehydrogenase, alkaline phosphates, β-glucosidase, amylase and arylesterase was observed in treated samples which are for conservation of soil health. These enzymes are involved in microbial oxidative activity, phosphate mineralization, carbon metabolism and degradation of complex compounds and their mobilization. Increased in enzyme activity after the treatments could be associated to improved microbial population in treated soil **[55]**. Dehydrogenases are generally essential enzymes and act as marker of dynamic biological action of the soil, involves in reductive activities of microbes. **Kumari et al. [56]** observed enhanced activities of FDA and alkaline phosphatase on the exposure of nanocompounds in Fenugreek. Our results recommend that combined application of nanogypsum and *Pseudomonas taiwanensis* is beneficial for plant and soil health as this treatment enhances bacterial population and enzyme activities of the soil. **Ju et al. [57]** reported that coinoculation of PGPR in soil enhanced urease, saccharase and β-glucosidase activity and improved microbial diversity in alfalfa in copper contaminated soil. **Du et al. [58]** reported toxic impact of zinc oxide nanoparticles on catalase and protease activity of the soil in wheat crop due to dissolution of ions.

As per the metagenomics study Chao1 and Shannon’s indices were reported significantly higher in treated soil than control. Comparison of metagenomic data clearly revealed that in the combined treatment (nanogypsum and bioinoculant), dominant bacterial phyla was *Proteobacteria*. Population of these phyla was higher in treated soil over control which is reported to play imperative role in metabolic performance of soil through their involvement in nutrient cycling **[59]**. Application of silver nanoparticles also augmented the plenty of *Proteobacteria* (about 30%) in treated soil over control **[60]**. In contrast, after the treatment of cerium oxide nanoparticles (nCeO) a decreased number of reads assigned to *Proteobacteria* was reported in activated sludge **[61]**. Occurrence of *Nitrospirae* in a healthy sample indicates good nitrification which results in enhanced nitrogen uptake by plants. Presence of *Flavobacterium*, *Sphinogomonas*, *Bacillus*, *Streptomyces*, *Planctomyces* and *Pseudomonas* sp. increases plant growth and improves soil fertility via mineral solubilization. Abundance of *Acidobacteria* is positively correlated with the availability of organic carbon and disease suppression **[62]**. **Sillen et al. [63]** observed that application of nanosilver increased the abundance of bacterial groups such as *Bacteroidetes* and *Acidobacteria*, which are widely recognized for their biocontrol potential, and decreased the population of *Planctomycetes* and *Actinobacteria*. Similarly, **Kibby and Strevett [64]** also reported that application of sulphate modified polystyrene nanomaterial increased the rhizospheric microbial population. Rarefaction curves are also used to measure the species richness for individual sample and highlight the resemblance or dissimilarity of bacterial diversity. Even distribution of microbial community within the treated soil can be depicted through steeper rarefaction curve. Overall results revealed that application of nanogypsum and bioinoculant improved physicochemical properties of the soil, bacterial counts and soil enzyme activities and microbial diversity of soil which helped in sustainable health improvement of maize crop. Nanogypsum can be applied as bioformulation to sustain the continued existence of bioinoculant for longer time and can offer an ecofriendly approach towards sustainable agriculture.

## Conclusion

Application of nanogypsum along with *Pseudomonas taiwanensis* significantly improved soil properties, especially microbial population and also expected to improve crop production. The underlying mechanisms responsible for improved microbial population in experimental soil under nanogypsum treatment included better nutrient management and water use efficiency. Nanogypsum positively supported growth and performance of bioinoculant and other valuable microbial population which thereafter supported plant health. Using a bioinoculant along with nanogypsum can be a better alternative to agrochemicals used in the agricultural practices. Metagenomic study of the treated soil revealed that application of nanogypsum was beneficial for the growth and survival of bacteria belonging to different phyla. A thorough understanding of mechanisms involved in the interactions between nanogypsum, soil and bioinoculant is needed under varied concentrations of nanocompound for enhanced crop production.

## Supporting information

S1 Table Physico-chemical properties of soil treated with nanogypsum and *Pseudomonas taiwanensis*. (DOCX)

S2 Table Sequencing parameters for control and treated soil DNA. (DOCX)

S1 Graphical abstract (TIF)

## References

[1] Almaghrabi OA, Abdelmoneim TS, Albishri HM, Moussa TA. Enhancement of maize growth using some plant growth promoting rhizobacteria (PGPR) under laboratory conditions. Life Science J. 2014; 11: 764–772.

[2] Zerrouk IZ, Rahmoune B, Khelifi L, Mounir K, Baluska F, Ludwig-Müller J. Algerian Sahara PGPR confers maize root tolerance to salt and aluminum toxicity via ACC deaminase and IAA. Acta Physiologiae Plantarum. 2019;41: 91. 10.1007/s11738-019-2881-2

[3] Glick BR. Plant Growth-Promoting Bacteria: mechanisms and applications. Hindawi Publishing Corporation, Scientifica. 2012. 10.6064/2012/963401 PMC3820493 24278762

[4] Ambrosini A, de Souza R, Passaglia LM. Ecological role of bacterial inoculants and their potential impact on soil microbial diversity. Plant and Soil. 2016; 400(1–2): 193–207. 10.1007/s11104-015-2727-7

[5] Baas P, Bell C, Mancini LM, Lee MN, Conant RT, Wallenstein MD. Phosphorus mobilizing consortium Mammoth P™ enhances plant growth. PeerJ. 2016; 4: e2121. 10.7717/peerj.2121 27326379 PMC4911952

[6] Chaudhary P, Sharma A. Response of Nanogypsum on the Performance of Plant Growth Promotory Bacteria Recovered from Nanocompound Infested Agriculture Field. Envi. and Ecol. 2019; 37 (1B): 363–372.

[7] Khati P, Chaudhary P, Gangola S, Sharma P. Influence ofNanozeolite on Plant Growth Promotory Bacterial Isolates Recovered from Nanocompound Infested Agriculture Field. Envi. and Ecol. 2019a; 37 (2): 521–527.

[8] Iavicoli I, Leso V, Beezhold DH, Shvedova AA. Nanotechnology in agriculture: Opportunities, toxicological implications, and occupational risks. Toxicology and Applied Pharmaco. 2017; 329: 96–111. 10.1016/j.taap.2017.05.025 28554660 PMC6380358

[9] Chakravarty D, Erande MB, Late DJ. Graphene quantum dots as enhanced plant growth regulators: effects on coriander and garlic plant*s*. J. of Science and Food Agriculture. 2015; 95: 2772–2778. 10.1002/jsfa.7106 25624024

[10] Aslani F, Bagheri S, Muhd Julkapli N, Juraimi AS, Hashemi FSG, Baghdadi A. Effects of engineered nanomaterials on plants growth: an overview. The Scientific World J. 2014. 10.1155/2014/641759 25202734 PMC4150468

[11] Chen L, Lee YB, Ramsier C, Bigham J, Slater B, Dick WA. Increased crop yield and economic return and improved soil quality due to land application of FGD-gypsum. In: Proceedings of the World of Coal Ash. Lexington, Ky. 2005.

[12] Kumar MS, Thiyageshwari S. Performance of Nano-Gypsum on Reclamation of Sodic soil. International J of Current Microbiol and Appl Sci. 2018; 6: 56–62.

[13] Parul, 2019. Application of conventional and metagenomic approaches to analyse the effect of nanocompounds and indigenous bioinoculants on the health of soil and *Zea mays*. Ph.D. Thesis. G.B.P.U.A & T, Pantnagar.

[14] Sekhon BS. Nanotechnology in agri-food production: an overview. Nanotechnology, Sci and appl. 2014; 7: 31. 10.2147/NSA.S39406 24966671 PMC4038422

[15] Liu R, Lal R. Potential of engineered nanoparticles as fertilizers for increasing agronomic productions. Sci of The Total Envi. 2015; 514: 131–139. 10.1016/j.scitotenv.2015.01.104 25659311

[16] Backer R, Rokem JS, Ilangumaran G, Lamont J, Praslickova D, Ricci E. et al. Plant growth-promoting rhizobacteria: context, mechanisms of action, and roadmap to commercialization of biostimulants for sustainable agriculture. Frontiers in plant Sci. 2018; 9: 1473. 10.3389/fpls.2018.01473 PMC6206271 30405652

[17] Feng Y, Cui X, He S, Dong G, Chen M, Wang J. et al. The role of metal nanoparticles in influencing arbuscular mycorrhizal fungi effects on plant growth. Environmental Sci & Technol. 2013; 47(16): 9496–9504. 10.1021/es402109n 23869579

[18] Smalla K, Wieland G, Buchner A, Zock A, Parzy J, Kaiser S. Bulk and rhizosphere soil bacterial communities studied by denaturing gradient gel electrophoresis: plant-dependent enrichment and seasonal shifts revealed. Applied and Environmental Microbiol. 2001; 67(10): 4742–4751. 10.1128/aem.67.10.4742-4751.2001 11571180 PMC93227

[19] Simonin M, Richaume A. Impact of engineered nanoparticles on the activity, abundance, and diversity of soil microbial communities: a review. Environmental Sci and Pollution Res. 2015; 1–14. 10.1007/s11356-015-4171-x 25647498

[20] Hiscox JD, Israelstam GF. A method for the extraction of chlorophyll from leaf tissue without maceration. Canadian J of Botany. 1979; 57(12): 1332–1334. 10.1139/b79-163.

[21] Kirk JT, Allen RL. Dependence of chloroplast pigment synthesis on protein synthesis: effect of actidione. Biochem and Biophysical Res Communications. 1965; 21(6): 523–530. 10.1016/0006-291x(65)90516-4 5879460

[22] Dubois KA, Gilles JK, Hamilton PA, Rebers FS. Calorimetric method for determination of sugars and related substances. Analytical Chem. 1956; 28(3):350–356. 10.1021/ac60111a017

[23] Bradford MM. A Rapid and Sensitive Method for the Quantitation of Microgram Quantities of Protein Utilizing the Principle of Protein-Dye Binding. Analytical Biochem. 1976; 72: 248–254. 10.1006/abio.1976.9999 942051

[24] Ainsworth EA, Gillespie KM. Estimation of total phenolic content and other oxidation substrates in plant tissues using Folin–Ciocalteu reagent. Nature Protocols. 2007; 2: 875–7. 10.1038/nprot.2007.102 17446889

[25] Chandlee JM, Scandalios JG. Analysis of variants affecting the catalase developmental program in maize scutellum. Theoretical Appl Genetics. 1984; 69: 71–7. 10.1007/BF00262543 24253626

[26] Mali PC, Vyas SP, Satish LL. Biochemical components of clusterbean genotypes in relation to bacterial blight. Indian Phytopathol. 1989; 42: 559–61.

[27] Giannopolitis CN, Ries SK. Superoxide dismutases I. Occurrence in higher plants. Plant Physiol. 1977; 59: 309–314. 10.1104/pp.59.2.309 16659839 PMC542387

[28] Schnurer J, Rosswall T. Fluorescein diacetate hydrolysis as a measure of total microbial activity in soil and litter. Applied and Environ. Microbiol.1982;43: 1256–1261. 10.1128/AEM.43.6.1256-1261.1982 16346026 PMC244223

[29] Casida L Jr, Klein D, Santoro T. Soil dehydrogenase activity. Soil Science. 1964;98: 371–377.

[30] Tabatabai MA, Bremner JM. Use of p-nitrophenyl phosphate for assay of soil phosphatise activity. Soil Biology and Biochem. 1969; 1(4): 301–307. 10.1016/0038-0717(69)90012-1

[31] Tabatabai MA. Soil enzymes. In: Weaver RW, Angle JS, Bottomley PS (eds) Methods of soil analysis, part 2. Microbiological and biochemical properties. Soil Science Society of America, Madison. 1994a; pp 775–833.

[32] Bernfeld P. Enzymes of starch degradation and synthesis. Advances in Enzymology and Related Subject of Biochem. 1951; 12: 379–428. 10.1002/9780470122570.ch7 14885023

[33] Nakamura T, Mochida K, Ozoe Y, Ukawa S, Sakai M, Mitsugi S. Enzymological properties of three soil hydrolases and effects of several pesticides on their activities. Journal of Pesticide Science (Japan). 1990; 15: 593–598.

[34] Mahakham W, Sarmah AK, Maensiri S, Theerakulpisut P. Nanopriming technology for enhancing germination and starch metabolism of aged rice seeds using phytosynthesized silver nanoparticles.Sci Rep. 2017; 7(1): 8263. 10.1038/s41598-017-08669-5 28811584 PMC5557806

[35] Shinde S, Paralikar P, Ingle AP, Rai M. Promotion of seed germination and seedling growth of *Zea mays* by magnesium hydroxide nanoparticles synthesized by the filtrate from *Aspergillus niger*. Arabian J. of Chemistry. 2018; 10.1016/j.arabjc.2018.10.001.

[36] Khati P, Chaudhary P, Gangola S, Bhatt P, Sharma A. Nanochitosan supports growth of *Zea mays* and also maintains soil health following growth. 3Biotech. 2017b; 7: 81. 10.1007/s13205-017-0668-y PMC5429309 28500403

[37] Khati P, Parul Bhatt P, Nisha Kumar R, Sharma A. Effect of nanozeolite and plant growth promoting rhizobacteria on maize. 3Biotech. 2018; 8: 141. 10.1007/s13205-018-1142-1 29484280 PMC5818361

[38] Agri U, Chaudhary P, Sharma A. In vitro compatibility evaluation of agriusable nanochitosan on beneficial plant growth-promoting rhizobacteria and maize plant. Natl. Acad. Sci. Lett. 2021; 10.1007/s40009-021-01047-w

[39] Chaudhary P, Khati P, Chaudhary A, Gangola S, Kumar R, Sharma A. Bioinoculation using indigenous *Bacillus* spp. improves growth and yield of *Zea* mays under the influence of nanozeolite. 3Biotech. 2021a; 11: 11. 10.1007/s13205-020-02561-2 PMC7778671 33442510

[40] Stepanova AN, Yun J, Likhacheva AV, Alonso JM. Multilevel interactions between ethylene and auxin in *Arabidopsis* roots. Plant Cell. 2007; 19: 2169–2185. 10.1105/tpc.107.052068 17630276 PMC1955696

[41] Qessaoui R, Bouharroud R, Furze JN, Aalaoui MEl, Akroud H, Amarraque A. et al. Applications of New Rhizobacteria *Pseudomonas* Isolates in Agroecology via Fundamental Processes Complementing Plant Growth. Sci. Reports. 2019; 9: 12832. 10.1038/s41598-019-49216-8 31492898 PMC6731270

[42] Chaudhary P, Sharma A, Chaudhary A, Khati P, Gangola S, Maithani D. Illumina based high throughput analysis of microbial diversity of rhizospheric soil of maize infested with nanocompounds and *Bacillus* sp. Applied Soil Ecology. 2021b; 159: 103836. 10.1016/j.apsoil.2020.103836.

[43] Hanan H, Latif Ghareib M, Tahon MA. Phytosynthesis of Silver Nanoparticles using Leaf Extracts from *Ocimum basilicum* and *Mangifira indica* and their Effect on some Biochemical Attributes of *Triticum aestivum*. Gesunde Pflanzen. 2017; 69: 39–46. 10.1007/s10343-017-0385-9

[44] Olanrewaju OS, Babalola OO. Bacterial Consortium for Improved Maize (*Zea mays* L.) Production. Microorganisms. 2019; 7(11): 519. 10.3390/microorganisms7110519 31683950 PMC6920993

[45] Urbonaviciute A, Samuoliene G, Sakalauskaite J, Duchovskis P, Brazaityte A, Siksnianiene JB. et al. The effect of elevated CO2 concentrations on leaf carbohydrate, chlorophyll contents and photosynthesis in radish. Polish Journal of Environmental Studies. 2006; 15: 921–925.

[46] Venkatachalam P, Priyanka N, Manikandan K, Ganeshbabu I, Indiraarulselvi P, Geetha N. et al. Enhanced plant growth promoting role of phycomolecules coated zinc oxide nanoparticles with P supplementation in cotton (*Gossypium hirsutum* L.) Plant Physiology and Biochem. 2017; 110: 118e127. 10.1016/j.plaphy.2016.09.004 27622847

[47] Chandra S, Chakraborty N, Dasgupta A, Sarkar J, Panda K, Acharya K. et al. Chitosan nanoparticles: A positive modulator of innate immune responses in plants. Sci Rep. 2015; 5: 15195. 10.1038/srep15195 26471771 PMC4607973

[48] Siddaiah NC, Keelara VHP, Niranjan RS, Venkataramana M, Vijai KG, Naveen K K. et al. Chitosan nanoparticles having higher degree of acetylation induce resistance against pearl millet downy mildew through nitric oxide generation. Sci Rep. 2018; 8: 2485. 10.1038/s41598-017-19016-z 29410438 PMC5802724

[49] Sandhya V, Ali SZ, Grover M. Effect of plant growth promoting *Pseudomonas* spp. on compatible solutes, antioxidant status and plant growth of maize under drought stress. Plant Growth Regul. 2010; 62: 21–30.

[50] García-Salamanca A, Molina-Henares MA, van Dillewijn P, Solano J, Pizarro-Tobías P, Roca A. Bacterial diversity in the rhizosphere of maize and the surrounding carbonate-rich bulk soil. Microbial Biotechnol. 2013; 6(1): 36–44. 10.1111/j.1751-7915.2012.00358.x 22883414 PMC3815383

[51] Ghosh A, Dey K, Bhowmick N, Medda PS, Dey AN. Reproductive Behaviour of Lemon (*Citrus limon Burm*.) Affected by Different Pruning Intensities and Integrated Nutrient Management under Various Growing Season. International J Current Microbiology Applied Sci. 2017; 6(4): 606–614.

[52] Chai H, Yao J, Sun J, Zhanf C, Liu W, Zhu M, et al. The effect of metal oxide Nanoparticles on functional bacteria and metabolic profiles in agricultural soil. Bulletin Envi. Contamination Toxicol. 2015; 94: 490–495. 10.1007/s00128-015-1485-9 25636440

[53] Kukreti B, Sharma A, Chaudhary P, Agri U, Maithani D. Influence of nanosilicon dioxide along with bioinoculants on *Zea mays* and its rhizospheric soil. 3Biotech. 2020; 10: 345. 10.1007/s13205-020-02329-8 32728512 PMC7374527

[54] Yang S, Xu Z, Wang R, Zhang Y, Yao F, Zhang Y. Variations in soil microbial community composition and enzymatic activities in response to increased N deposition and precipitation in Inner Mongolian grassland. Applied Soil Ecology. 2017; 119: 275–285. 10.1016/j.apsoil.2017.06.041

[55] Timmusk S, Seisenbaeva G, Behers L. Titania (TiO2) nanoparticles enhance the performance of growth-promoting rhizobacteria. Sci. Rep. 2018; 8: 617. 10.1038/s41598-017-18939-x 29330479 PMC5766586

[56] Kumari S, Sharma A, Chaudhary P, Khati P. Management of plant vigor and soil health using two agriusable nanocompounds and plant growth promotory rhizobacteria in Fenugreek. 3Biotech. 2020; 10: 461. 10.1007/s13205-020-02448-2 33088658 PMC7532980

[57] Ju W, Liu L, Jin X, Duan C, Cui Y, Wang J, et al. Co-inoculation effect of plant-growth-promoting rhizobacteria and *Rhizobium* on EDDS assisted phytoremediation of Cu contaminated soils, Chemosphere. 2020. 10.1016/j.chemosphere.2020.126724 32334248

[58] Du W, Sun Y, Ji R, Zhu J, Wu J, Guo H. TiO2 and ZnO nanoparticles negatively affect wheat growth and soil enzyme activities in agricultural soil. J. Environ. Monit. 2011; 13: 822–828. 10.1039/c0em00611d 21267473

[59] Mashiane RA, Ezeokoli OT, Adeleke RA, Bezuidenhout CC. Metagenomic analyses of bacterial endophytes associated with the phyllosphere of a Bt maize cultivar and its isogenic parental line from South Africa. World J. of Microbiol. 2018. 10.1007/s11274-017-2249-y 28341909

[60] Chavan S, Nadanathangam V. Effects of nanoparticles on plant growth-promoting bacteria in Indian agricultural soil. Agronomy. 2019; 9: 140. 10.3390/agronomy9030140

[61] Kamika I, Tekere M. Impacts of cerium oxide nanoparticles on bacterial community in activated sludge. AMB Express. 2017; 7(1): 63. 10.1186/s13568-017-0365-6 28299750 PMC5352701

[62] Khati P, Sharma A, Chaudhary P, Singh AK, Gangola S, Kumar R. High- throughput sequencing approach to access the impact of nanozeolite treatment on species richness and evens of soil metagenome. Biocatalysis and Agri. Biotech. 2019b; 20: 101249. 10.1016/j.bcab.2019.101249.

[63] Sillen WMA, Thijs S, Abbamondi GR, Roche RD, Weyens N, White JC et al. Nanoparticle treatment of maize analyzed through the metatranscriptome: compromised nitrogen cycling, possible phytopathogen selection and plant hormesis. Microbiome. 2020; 8: 127. 10.1186/s40168-020-00904-y 32907632 PMC7488162

[64] Kibbey TCG, Strevett KA. The effect of nanoparticles on soil and rhizosphere bacteria and plant growth in lettuce seedlings. Chemosphere. 2019; 221: 703–707. 10.1016/j.chemosphere.2019.01.091 30669112

