# Supplementary material for: Cultivable and metagenomic approach to study the combined impact of nanogypsum and Pseudomonas taiwanensis on maize plant health and its rhizospheric microbiome
Source: PLoS One. 2021 Apr 26;16(4):e0250574. doi: 10.1371/journal.pone.0250574 (PMC8075249; doi:10.1371/journal.pone.0250574)
Supplement: S1 Table — (DOCX) [file pone.0250574.s001.docx]

**S1 Table.** Physico-chemical properties of soil treated with Nanogypsum and *Pseudomonas taiwanensis*

| **Treatments** | Soil pH | OC ^a^ | | | | | APH ^b^ | | | | | APT ^c^ | | | | AN ^d^ | | | NN ^e^ | | | |
| --- | --- | --- | --- | --- | --- | --- | --- | --- | --- | --- | --- | --- | --- | --- | --- | --- | --- | --- | --- | --- | --- | --- |
| **Scale** |  | L | ML | M | MH | H | B | L | M | MH | H | L | M | H | VH | L | M | H | VL | L | M | H |
| **AC** | 7.61 |  |  |  |  |  |  |  |  |  |  |  |  |  |  |  |  |  |  |  |  |  |
| **PC1** | 7.76 |  |  |  |  |  |  |  |  |  |  |  |  |  |  |  |  |  |  |  |  |  |
| **NG** | 7.77 |  |  |  |  |  |  |  |  |  |  |  |  |  |  |  |  |  |  |  |  |  |
| **PC1+NG** | 7.80 |  |  |  |  |  |  |  |  |  |  |  |  |  |  |  |  |  |  |  |  |  |

**As per “K054 soil testing Kit; Himedia Laboratories Pvt Ltd India “Black boxes show results.**

**a**: OC, organic carbon (Kgha^-1^) L: low (0.1- 0.3); ML: Medium low (0.300-0.500); M: Medium (0.500-0.750); MH: Medium High (0.750-1.00); H: High (1.00-1.50) **b**: APH, Available phosphate as P_2_O_5_(Kgha^-1^) B: Blank; L: Low (<22); M: Medium (22-56); MH: Medium High (56-73); H: High (<73)

**c**: APT, Available potassium as K_2_O (Kgha^-1^) L: Low (>112); M: Medium (112- 280); H: High (280-392); VH: Very High (<393)

**d**: AN, Ammonical nitrogen (Kgha^-1^) L: Low (about 15); M: Medium (about 73); H: High (about 202)

**e**: NN, Nitrate nitrogen (Kgha^-1^): VL: Very low (about 04); L: Low (about 10); M: Medium (about 20); H: High (about 50)
