# Supplementary material for: Cultivable and metagenomic approach to study the combined impact of nanogypsum and Pseudomonas taiwanensis on maize plant health and its rhizospheric microbiome
Source: PLoS One. 2021 Apr 26;16(4):e0250574. doi: 10.1371/journal.pone.0250574 (PMC8075249; doi:10.1371/journal.pone.0250574)
Supplement: S2 Table — (DOCX) [file pone.0250574.s002.docx]

**S2 Table.** Sequencing parameters for control and treated soil DNA

| **Sequencing parameters** | **Control soil Replicate 1** | **Control soil Replicate 2** | **Treated soil Replicate 1 (PC1NG)** | **Treated soil Replicate 2 (PC1NG)** |
| --- | --- | --- | --- | --- |
| Total number of bases | 169154776 | 169154776 | 114483544 | 114483544 |
| Total number of reads | 280988 | 280988 | 190172 | 190172 |
| % bases>=Q20 | 85 | 85 | 86 | 86 |
| % bases>=Q30 | 88 | 88 | 88 | 88 |
| Average sequence length | 301 | 301 | 301 | 301 |
| GC content % | 57.64 | 57.64 | 56.28 | 57 |
