# Supplementary figures and images for: Cultivable and metagenomic approach to study the combined impact of nanogypsum and Pseudomonas taiwanensis on maize plant health and its rhizospheric microbiome
Source: PLoS One. 2021 Apr 26;16(4):e0250574. doi: 10.1371/journal.pone.0250574 (PMC8075249; doi:10.1371/journal.pone.0250574)

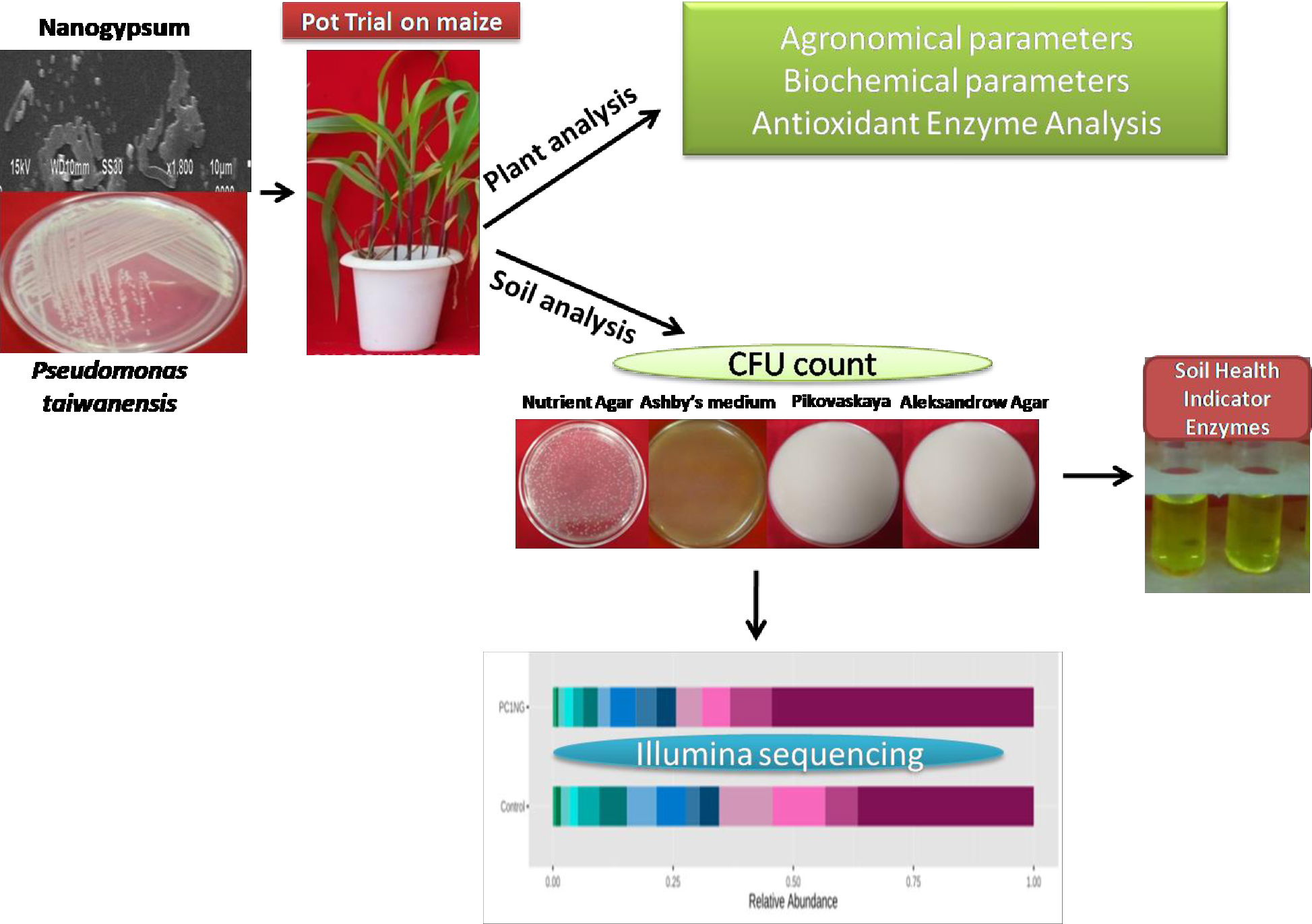

Supplement: S1 Graphical abstract — (TIF) [file pone.0250574.s003.tif]
